# Fractal zone plate beam based optical tweezers

**DOI:** 10.1038/srep34492

**Published:** 2016-09-28

**Authors:** Shubo Cheng, Xinyu Zhang, Wenzhuo Ma, Shaohua Tao

**Affiliations:** 1 School of Physics and Electronics, Central South University, Changsha 410083, China; 2 Hunan Key Laboratory of Super Microstructure and Ultrafast Process, Central South University, Changsha 410083, China

## Abstract

We demonstrate optical manipulation with an optical beam generated by a fractral zone plate (FZP). The experimental results show that the FZP beam can simultaneously trap multiple particles positioned in different focal planes of the FZP beam, owing to the multiple foci and self-reconstruction property of the FZP beam. The FZP beam can also be used to construct three-dimensional optical tweezers for potential applications.

Optical trap^1^ formed by focusing a laser beam into a diffraction-limited spot with a high Numerical Aperture (NA) objective has been widely used to trap various types of particles such as transparent silica or polymer spheres, dielectric-covered metallic microspheres, and so on^2^,^3^,^4^,^5^. As an alternative to trapping multiple particles, a single beam with multiple traps can be generated by using holographic optics^6^. For example, Melville *et al.*^7^ demonstrated trapping of particles in three-dimensional (3D) space via time-sharing and lensed holograms. Specific beam shaping algorithms were also used to form 3D structure of particles^8^,^9^,^10^,^11^. Although a single beam designed by a beam shaping algorithm can generate multiple traps in 3D space, distortion made by reflection, refraction, or absorption of a trapped particle would affect the subsequent propagation of the beam. To trap multiple particles separately distributed in 3D space, one can utilize a beam with tailorable 3D intensity distribution and the self-reconstruction property^12^,^13^, which is the pre-requirement for the trapping of multiple particles along the propagation axis of the beam. A few beams, e.g., Bessel beams, Airy beams, and others^14^,^15^,^16^,^17^,^18^, were found to have the self-reconstruction property. The Bessel beams and the Airy beams were used to simultaneously trap multiple particles^12^,^19^,^20^,^21^, but positions of the trapped particles in 3D space could not be customized owing to the non-diffracting intensity profiles of the beams in the propagation direction.

Recently, fractal zone plates (FZPs) were proposed to form two-dimensional photonic structures^22^. The axial irradiance of an FZP is characterized with the fractal profile versus the square of the radial coordinate and the multiple foci with internal fractal property along the optical axis^23^. The fractal pattern has the self-similarity when the FZP is illuminated with a plane wave^24^. Besides having one main focus and other weaker foci, i.e., the so-called major foci, an FZP beam also has many subsidiary foci around the main focus and major foci along the optical axis^25^. Owing to the unique diffraction property, the FZP beams have potential applications in the research fields such as 3D optical trapping, optical imaging, and so on^25^,^26^. Moreover, as focal lengths of multiple foci provided by an FZP beam are determined by the structural parameters of the FZP, the axial distances between adjacent foci of the beam can be finely controlled with a fractional number of the segments of the fractal structure^25^. Thus, with the FZP beams one can realize not only the simultaneous trapping of multiple particles in different planes but also the customized trapping locations of trapped particles in 3D space. Athough optical trapping with a beam generated by the Devil’s lens characterized with a fractal structure was realized^27^, the simultaneous manipulation of particles at different axial positions has not been reported.

In this letter we will demonstrate the capability of an FZP beam in constructing 3D optical tweezers. The unique diffraction property of the FZP beam such as the self-reconstruction is critical for the realization of the 3D manipulation, so the main focus and the surrounding subsidiary foci of the FZP beam can be used to separately trap and manipulate particles at different positions in 3D space.

## Results

### The axial irradiance of FZP

An FZP comprises a series of pupils that holds the fractal structure^22^. The FZPs can be constructed from conventional Fresnel zone plates in some cases. The FZP beam possesses multiple foci with internal fractal property along the optical axis^22^,^23^. An expression for the transmittance of a single FZP can be found in ref. 22, and the transmittance comprises 0 and 1, meaning that the corresponding zone of the FZP is opaque or transparent to the incident beam. The focal length of the main focus of an FZP beam can be expressed by Eq. (1)^23^,


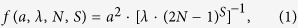


where *a* is the radius of the FZP, *λ* is the wavelength of the illuminating light, *N* is the number of the segments forming the fractal structure, and *S* is the fractal stage.

**Figure 1:**
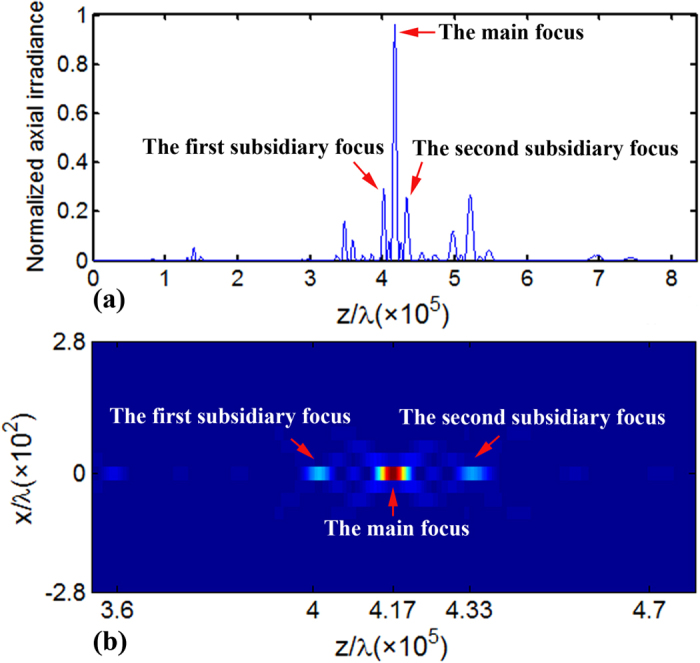
(**a**) Axial irradiance of the FZP with *N* = 3 and *S* = 3, and (**b**) the calculated intensity distribution of the FZP in the *x*_−_*z* plane, where the main focus and the two additional subsidiary foci are illustrated.

As phase-only diffractive optical elements have higher diffraction efficiency than the amplitude-only counterparts, the transmittance values of 0 and 1 of the FZP will be replaced with phase steps of 0 and π, respectively^28^. In Eq. (1), for instance, when the FZP is illuminated with a collimated laser beam with a wavelength of 532 *nm*, the focal length of the main focus of the FZP with *N* = 3 and *S* = 3 is *f* = 222 *mm*. Propagation of the FZP beam with *N* = 3 and *S* = 3 in free space is simulated by using the angular spectrum of the plane wave method. The axial irradiance of the FZP is shown in Fig. 1(a), where multiple foci with internal fractal properties along the *z* direction can be observed, and the main focus has the highest intensity and a distance of ~222 *mm* from the FZP. Futhermore, the additional foci surrounding the main focus are at distances of ~213 *mm* and 230 *mm*, respectively. Figure 1(b) shows the calculated intensity distributions of the FZP beam in the x-z plane. In Fig. 1 the peaks around the main focus are marked as the first and second subsidiary foci, respectively. As the FZP beams are found to have the self-reconstruction property^24^, the trapped particles in an FZP beam will not affect the subsequent propagation of the beam. The beam generated with the FZP with *N* = 3 and *S* = 3 will be used to trap particles in the experiments. Specifically, the main focus and the two subsidiary foci shown in Fig. 1 will be employed in the trapping.

**Figure 2:**
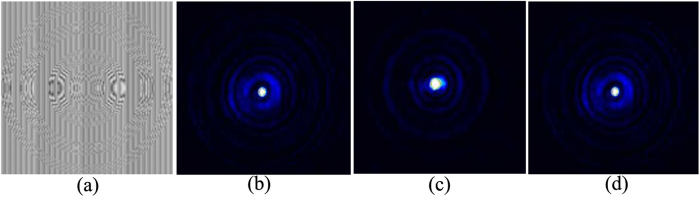
(**a**) Phase distribution of the FZP of *N* = 3 and *S* = 3, and (**b**–**d**) CCD-captured intensity distributions of the first subsidiary focus, the main focus, and the second subsidiary focus of the FZP beam at propagating distances of 21 *cm*, 22 *cm*, and 23 *cm*, respectively.

In the trapping experiments polystyrene beads with a diameter of about 3 *μm* were immersed in deionized water of refractive index of 1.33 and used as the manipulating objects. The binary phase-only FZP with *N* = 3 and *S* = 3 was loaded onto a spatial light modulator (SLM) to generate the FZP beam. We had added phase distribution of a blazed grating to the phase-only FZP for an off-axis output. The computer-generated hologram is shown in Fig. 2(a). The CCD-captured intensity distributions of the first subsidiary focus, the main focus, and the second subsidiary focus are shown in Fig. 2(b–d), respectively. The propagating distances of the beam for Fig. 2(b–d) are 21 *cm*, 22 *cm*, and 23 *cm*, respectively. The experimental intensity distributions in Fig. 2(b–d) are in accordance with those of the foci shown in Fig. 1. Obviously, the main focus shown in Fig. 2(c) has the highest intensity.

### Optical trapping and manipulating

**Figure 3:**
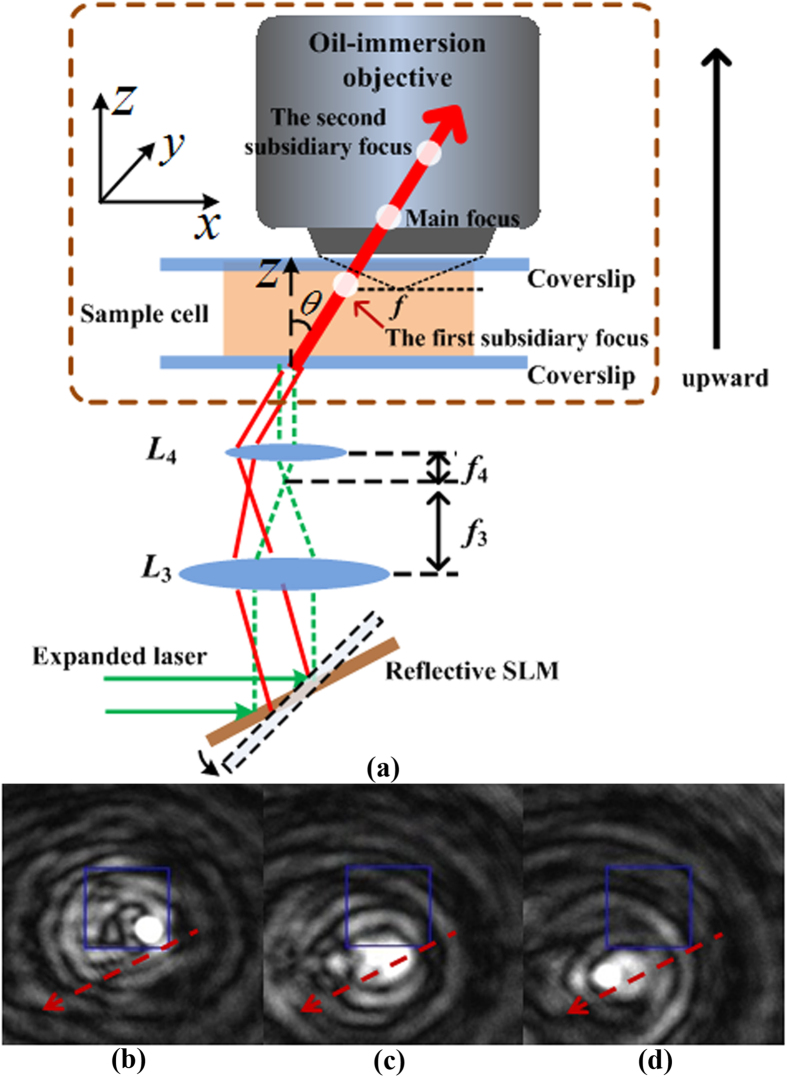
(**a**) Schematic of the experimental setup, and (**b**–**d**) captured CCD frames of intensity distributions of the first subsidiary focus, the main focus, and the second subsidiary focus, respectively. The dashed arrows in (**b**–**d**) represent the shifting directions of the foci with synchronous shifts of the sample cell and the objective.

In order to conveniently observe the 3D trapping of particles in multiple focal planes in the experiment, we arrange the optical path shown in Fig. 3(a), where the beam enters the sample cell at a small oblique angle. In Fig. 3(a) the dashed green line represents the collinear arrangement of optical components, and the red solid line shows the configuration of our trapping experiment. Figure 3(a) shows that the focus of the objective locates just in the sample cell and the first subsidiary focus is on the focal plane of the objective, so the first subsidiary focus in the cell can be clearly observed. The observed first subsidiary focus in the viewfield of the CCD camera is shown in the right bottom of the square exposure region in Fig. 3(b). It is worth mentioning that the relative distance between the objective and the sample cell was fixed in the optical trapping. When the sample cell and the objective is shifted upward synchronously about 180 *μm*, the main focus shown in Fig. 3(c) can be observed clearly in the viewfield. Although the beam has been shifted relatively toward the bottom of the sample cell, a dim image of the first subsidiary focus on the right of the main focus can still be observed in Fig. 3(c). The sample cell and the objective are further shifted upward synchronously until the second subsidiary focus appears clearly. The CCD-captured image of the second subsidiary focus is shown in Fig. 3(d). Similarly, a dim image of the main focus exists on the right of the second subsidiary focus. From Fig. 3(b–d) we can also observe that the locations of the three foci shift toward a fixed direction when the sample cell and the objective shift upward synchronously. The dashed arrows in Fig. 3(b–d) represent the shifting direction of the foci in the viewfield. Obviously, the displacement of the foci in the viewfield is resultant from the oblique incidence of the beam ray. The oblique incidence makes the observation of different particles in multiple planes more convenient. Compared with the two subsidiary foci in the CCD viewfield, the captured main focus shown in Fig. 3(c) has the highest intensity. Furthermore, we can estimate the incident angle of the beam ray on the sample cell from the displacements of the foci in the viewfield. For instance, when the first subsidiary focus is observed clearly in the viewfield and then the sample cell and the objective is shifted upward synchronously about *l μm*, the second subsidiary focus can be observed clearly. If the second subsidiary focus shifts a distance of *d μm* from the first subsidiary focus in the viewfield, the incident angle of the FZP beam on the sample cell can be expressed approximately by arctan(*d*/*l*).

**Figure 4:**
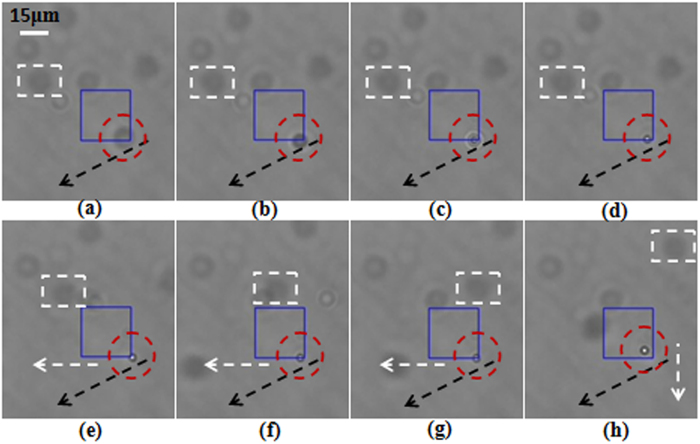
(**a**–**d**) CCD-captured frames showing that a particle highlighted with a dashed circle is attracted to the first subsidiary focus, and (**e**–**h**) CCD-captured frames showing that the trapped particle highlighted with a dashed circle is moved horizontally with the shifting focus.

**Figure 5:**
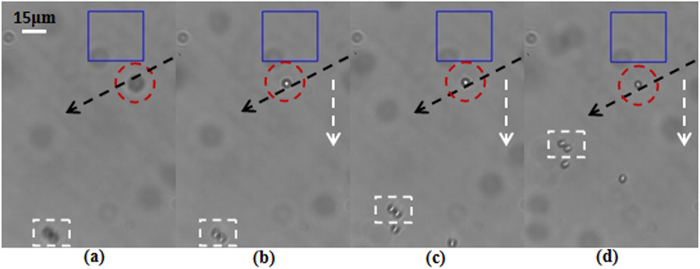
(**a**–**d**) CCD-captured frames showing that a particle highlighted with a dashed circle is trapped and then moved horizontally with the main focus of the FZP beam.

**Figure 6:**
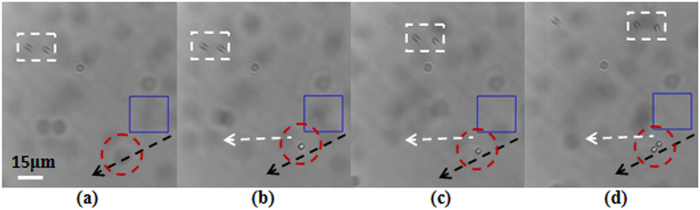
(**a**–**d**) CCD-captured frames showing that a particle highlighted with a dashed circle is trapped and then moved horizontally with the the second subsidiary focus of the FZP beam.

In the trapping experiment, the laser power was set to 800 *mW*. In the beginning the first subsidiary focus of the FZP beam was located in the focal plane of the objective. A polystyrene bead in the bottom of the sample cell was attracted toward the first subsidiary focus and then trapped stably. Figure 4(a–d) show the sequential CCD-captured frames demonstrating that a polystyrene bead is attracted toward the first subsidiary focus and then trapped stably. Figure 4(e–h) show the frames of the trapped bead moving horizontally with the first subsidiary focus. Then we shifted the sample cell and the objective upward synchronously and used the main and the second subsidiary foci to trap polystyrene beads, respectively. The video frames of the trapped particles moving horizontally under the forces of the main focus and the second subsidiary foci are shown in Fig. 5(a–d) and Fig. 6(a–d), respectively. In the frames the beads highlighted with the dashed rectangles and dashed circles are the reference particle and the trapped particle, respectively. The black dashed arrows are used as references for convenient observation of the shifting trajectory. The white dashed arrows represent the moving direction of the trapped particles. Hence, we can observe from Figs 4, 5, 6 that all the foci including the main focus and the two subsidiary foci of the FZP beam are capable of manipulating particles stably.

### 3D trapping of microparticles

**Figure 7:**
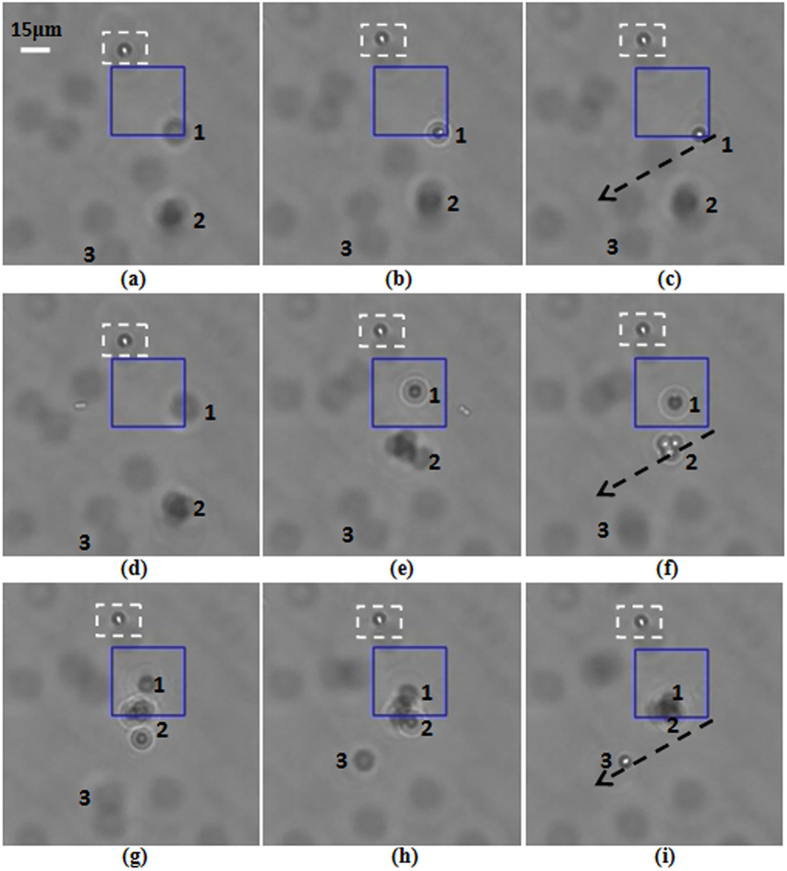
Experimental demonstration of simulataneous trapping with three foci of the FZP beam. (**a**–**c**) Trapping of the particle marked as 1 with the first subsidiary focus, (**d**–**f**) trapping of a cluster of particles marked as 2 with the main focus, and (**g**–**i**) trapping of the particle marked as 3 with the second subsidiary focus and the simulataneous trapping of the particles marked as 1 and 2 with the main focus, respectively.

As an FZP beam possesses multiple foci^22^, 3D trapping of particles can be realized with the beam. In the experiment the sample cell and the objective were shifted upward synchronously until the first subsidiary focus was observed clearly in the viewfield of the CCD camera. The observed first subsidiary focus is shown in Fig. 3(b). Then the sample cell moved horizontally until particles appeared in the viewfield. When the laser power was about 800 *mW*, the polystyrene bead marked as 1 was trapped by the first subsidiary focus, and the trapping images are shown in Fig. 7(a–c). The particles highlighted with the white dashed rectangles are used as the reference. When particle 1 had been trapped by the first subsidiary focus, the sample cell and the objective were shifted slowly upward synchronously. In the mean time we observed that particle 1 trapped by the first subsidiary focus was shifted toward the bottom of the sample cell with the first subsidiary focus, and then the CCD images of the trapped particle 1 became blurred. The trapping images are shown in Fig. 7(c,d). When the sample cell and the objective were shifted upward synchronously until the main focus appeared on the focal plane of the objective, a cluster of particles marked as 2 was trapped. The trapping images are shown in Fig. 7(d–f). Then the sample cell and the objective were further shifted upward synchronously, we observed that the cluster of trapped particles was shifted toward the bottom of the sample cell with the main focus, and particle 1 trapped by the first subsidiary focus was also attracted to the main focus for the higher intensity of the main focus. CCD images of particle 1 trapped by the main focus are shown in Fig. 7(f–h). The sample cell and the objective were further shifted upward synchronously until the second subsidiary focus appeared on the focal plane of the objective. At that time the particle marked as 3 was trapped by the second subsidiary focus. The trapping images are shown in Fig. 7(g–i). From Fig. 7(i) we can find that the cluster of particles is still trapped by the main focus in the bottom of the sample cell. Due to limited depth of the sample cell and short work distance of the objective, when the second subsidiary focus is located on the focal plane of the objective, the first subsidiary focus has moved out of the sample cell. Nonetheless, we can observe simulatnaneous trapping of particles by the second subsidiary focus and the main focus in the viewfield from Fig. 7(i). Hence, particles can be manipulated with the multiple foci in different planes. The simultaneous trappings in a row of the foci are attributed to the self-reconstruction property of the beam. In the trapping experiments, the distance between the trapping positions of the first subsidiary focus and the second focus in the viewfield of the CCD camera was about 25 *μm*. When the first subsidiary focus appeared clearly in the viewfield, the sample cell and the objective were shifted upward synchronously about 380 *μm* until the second subsidiary focus appeared. Thus, the incident angle on the sample cell was about 0.066 *radian*.

## Conclusion

An FZP beam based optical tweezers was demonstrated. In the experiment the main focus and two adjacent subsidiary foci of the FZP beam were used to trap polystyrene beads. The experimental results showed that the three foci could manipulate particles stably and independently. Furthermore, the second subsidiary focus and the main focus trapped different particles in the two respective focal planes simultaneously, owing to the self-reconstruction property of the beam. The results proved that the FZP beam could be employed to construct 3D optical tweezers to form complex particle structures or implement precise optical manipulations.

## Methods

**Figure 8:**
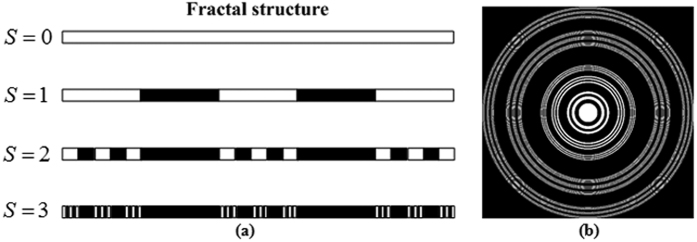
(**a**) Schematic of the one-dimensional fractal structure based on the Cantor sets, and (**b**) the associated FZP generated from the one-dimensional fractal structure with *S* = 3.

As an example, the construction procedure of a regular Cantor fractal is shown in Fig. 8(a)^23^. In the first stage, i.e., number of the fractal stage is *S* = 1, the segment is divided into an odd number of segments 2*N*−1 and the segments in the even-number positions are removed. For the remaining *N* segments at the first stage, the “slicing and removing” process is repeated in the subsequent stages. Figure 8(b) shows the associated FZP generated from the fractal structure with *S* = 3.

**Figure 9:**
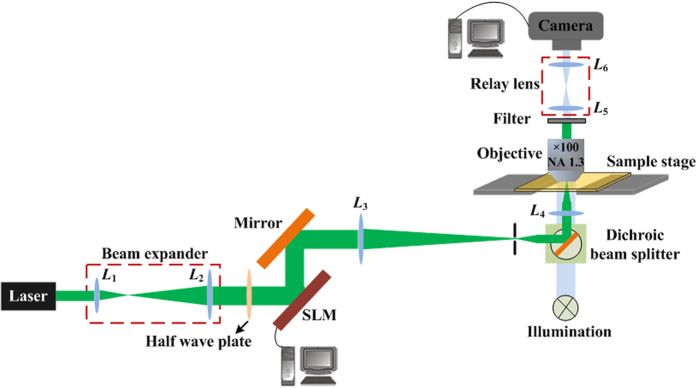
Schematic of the FZP beam based optical tweezers.

Optical trapping experiments were implemented with the presented FZP beam. The optical tweezers system based on an FZP beam is shown in Fig. 9. A Gaussian beam was emitted from an optically pumped semiconductor laser with maximum output power of 1 *W*, expanded with lenses *L*_1_ and *L*_2_ (*f*_1_ = 30 *mm* and *f*_2_ = 300 *mm*), and then projected onto the SLM (BNS, XY Nematic Series, 512 × 512 pixels, phase type, pixel pitch = 15 *μm*) screen. A half-wave retardation plate was inserted into the optical path to change the orientation of the polarization of the beam incident to the SLM^29^. The reconstructed FZP beam from the SLM was further reduced with lenses *L*_3_ and *L*_4_ (*f*_3_ = 750 *mm* and *f*_4_ = 30 *mm*) into a beam with an about 10 *μm* diameter–sized spot in the plane of the main focus. The imaging system viewing the sample stage comprised a 100× Olympus oil-immersion objective lens (N.A. 1.3), a telescope (*f*_5_ = 40 *mm* and *f*_6_ = 40 *mm*), a white-light light emission diode, and a CCD camera.

## Additional Information

**How to cite this article**: Cheng, S. *et al.* Fractal zone plate beam based optical tweezers. *Sci. Rep.* **6**, 34492; doi: 10.1038/srep34492 (2016).

## References

[b1] Ashkin A., Dziedzic J. M., Bjorkholm J. E. & Chu S. Observation of a single-beam gradient force optical trap for dielectric particles. Opt. Lett. 11, 288–290 (1986). 19730608 10.1364/ol.11.000288

[b2] Molloy J. E. & Padgett M. J. Lights, action: optical tweezers. Contemp. Phys. 43, 241–258 (2002).

[b3] Tao S. H., Yuan X. C. & Lin J. Fractional optical vortex beam induced rotation of particles. Opt. Express 13, 7726–7731 (2005). 19498800 10.1364/opex.13.007726

[b4] Cheng S. B., Tao S. H., Zhou C. H. & Wu L. Optical trapping of a dielectric-covered metallic microsphere. J. Opt. 17, 105613 (2015).

[b5] Gongora J. & Fratalocchi A. Optical force on diseased blood cells: Towards the optical sorting of biological matter. Opt. Laser Eng. 76, 40–44 (2016).

[b6] Hayasaki Y. *et al.* Optical manipulation of microparticles using diffractive optical elements. Proc. SPIE 2778, 229–230 (1996).

[b7] Melville H. & Milne G. F. Optical trapping of three-dimensional structures using dynamic holograms. Opt. Express 11, 3562–3567 (2003). 19471491 10.1364/oe.11.003562

[b8] Leach J. *et al.* 3D manipulation of particles into crystal structures using holographic optical tweezers. Opt. Express 12, 220–226 (2004). 19471528 10.1364/opex.12.000220

[b9] Sinclair G. *et al.* Interactive application in holographic optical tweezers of a multi-plane Gerchberg-Saxton algorithm for three-dimensional light shaping. Opt. Express 12, 1665–1670 (2004). 19474992 10.1364/opex.12.001665

[b10] Sinclair G. *et al.* Assembly of 3-dimensional structures using programmable holographic optical tweezers. Opt. Express 12, 5475–5480 (2004). 19484108 10.1364/opex.12.005475

[b11] Benito D. C. *et al.* Constructing 3D crystal templates for photonic band gap materials using holographic optical tweezers. Opt. Express 16, 13005–13015 (2008). 18711539 10.1364/oe.16.013005

[b12] Bouchal Z., Wagner J. & Chlup M. Self-reconstruction of a distorted nondiffracting beam. Opt. Commun. 151, 207–211 (1998).

[b13] Cox A. J. & D’Anna J. Constant-axial-intensity nondiffracting beam. Opt. Lett. 17, 232–234 (1992). 19784285 10.1364/ol.17.000232

[b14] Garces-Chavez V., McGloin D., Melville H., Sibbett W. & Dholakia K. Simultaneous micromanipulation in multiple planes using a self-reconstructing light beam. Nature 419, 145–147 (2002). 12226659 10.1038/nature01007

[b15] McGloin D., Garcés-Chávez V. & Dholakia K. Interfering Bessel beams for optical micromanipulation. Opt. Lett. 28, 657–659 (2003). 12703932 10.1364/ol.28.000657

[b16] Tao S. H. & Yuan X. C. Self-reconstruction property of fractional Bessel beams. J. Opt. Soc. Am. A 21, 1192–1197 (2004). 10.1364/josaa.21.001192 15260251

[b17] Broky J., Siviloglou G. A., Dogariu A. & Christodoulides D. N. Self-healing properties of optical Airy beams. Opt. Express 16, 12880–12891 (2008). 18711527 10.1364/oe.16.012880

[b18] Anguiano-Morales M., Martínez A., Iturbe-Castillo M. D., Chávez-Cerda S. & Alcalá-Ochoa N. Self-healing property of a caustic optical beam. Appl. Opt. 46, 8284–8290 (2007). 18059670 10.1364/ao.46.008284

[b19] Arlt J., Garces-Chavez V., Sibbett W. & Dholakia K. Optical micromanipulation using a Bessel light beam. Opt. Commun. 197, 239–245 (2001).

[b20] Zheng Z., Zhang B. F., Chen H., Ding J. P. & Wang H. T. Optical trapping with focused Airy beams. Appl. Opt. 50, 43–49 (2011). 21221158 10.1364/AO.50.000043

[b21] Zhang P. *et al.* Trapping and guiding microparticles with morphing autofocusing Airy beams. Opt. Lett. 36, 2883–2885 (2011). 21808346 10.1364/OL.36.002883

[b22] Saavedra G., Furlan W. D. & Monsoriu J. A. Fractal zone plates. Opt. Lett. 28, 971–973 (2003). 12836749 10.1364/ol.28.000971

[b23] Monsoriu J. A., Furlan W. D. & Saavedra G. Focusing light with fractal zone plates. Recent Res. Devel. Opt. 5 (2005).

[b24] Davis J. A., Ramirez L., Martín-Romo J. A., Alieva T. & Calvo M. L. Focusing properties of fractal zone plates: experimental implementation with a liquid-crystal display. Opt. Lett. 29, 1321–1323 (2004). 15233422 10.1364/ol.29.001321

[b25] Tao S. H., Yang B. C., Xia H. & Yu W. X. Tailorable threedimensional distribution of laser foci based on customized fractal zone plates. Laser Phys. Lett. 10, 035003 (2013).

[b26] Furlan W. D., Saavedra G. & Monsoriu J. A. White-light imaging with fractal zone plates. Opt. Lett. 32, 2109–2111 (2007). 17671552 10.1364/ol.32.002109

[b27] Pu J. & Jones P. H. Devil’s lens optical tweezers. Opt. Express 23, 8190–8199 (2015). 25968658 10.1364/OE.23.008190

[b28] Tao S. H., Yuan X.-C., Niu H. B. & Peng X. Dynamic optical manipulation using intensity patterns directly projected by a reflective spatial light modulator. Rev. Sci. Instrum. 76, 056103 (2005).

[b29] Tao S. H., Yuan X. C., Lin J. & Burge R. E. Sequence of focused optical vortices generated by a spiral fractal zone plate. Appl. Phys. Lett. 89, 031105 (2006).

